# Toward establishing a worldwide net of canine biobanks

**DOI:** 10.18632/aging.203961

**Published:** 2022-03-17

**Authors:** Sára Sándor, Silvan Urfer, Enikő Kubinyi

**Affiliations:** 1 Senior Family Dog Project, Department of Ethology, ELTE Eötvös Loránd University, Budapest, Hungary; 2 MTA-ELTE Lendület "Momentum" Companion Animal Research Group, Budapest, Hungary; 3 Dog Aging Project, Department of Laboratory Medicine and Pathology, University of Washington, Seattle, WA 98195, USA

**Keywords:** dog, biobank, aging, ccd

Companion dogs have run a spectacular course as a newly emerging model system to study complex phenomena, like aging and related pathologies, where studies in laboratory model organisms have often shown low translatability to humans. This limitation is pronounced in the case of human dementia, as canonical model animals – including rodents – do not naturally develop any homologous pathology. Dogs, in contrast, can be susceptible to age-related neuropathological conditions, including a serious form of cognitive decline, termed Canine Cognitive Dysfunction (CCD). CCD bears several similarities with human Alzheimer’s disease – the most common form of age-related dementia – both in symptoms and neural pathology [1]. Importantly, these pathologies develop within a shorter time frame throughout the life course of dogs, as their average lifespan is roughly seven times shorter than that of humans. Hence, dogs could represent a solution to study the genetic and environmental risk factors of dementia in a natural population, which is exposed to similar stressors as the people, whom they live with as companions. Importantly, these animals can also be involved in pre-clinical trials to test preventive interventions and therapies within a more convenient time frame than would be allowed by human studies [2,3].

Not surprisingly, there is an increasing number of aging studies investigating specific companion dog populations [4]. However, to model human populations, data from companion dogs representing a wider spectrum of genetic and environmental variability would be the most advantageous [2]. Initiatives, like the Senior Family Dog Project in Hungary and the Dog Aging Project in the US have been set to recruit privately owned companion dog cohorts for long-term follow-up to gather behavioral and medical data throughout their life course. However, obtaining biological samples - e.g., muscle or brain tissue - for molecular and histological research is more challenging than in the case of laboratory animals, as there are more ethical regulations surrounding invasive collection methods in companion dogs. While the same issue has been addressed by large-scale medical databases and biobanking services in human medical research, similar approaches have only recently been initiated in the case of companion dogs.

The Canine Brain and Tissue Bank (CBTB) at the Eötvös Loránd University in Hungary led by Dr Enikő Kubinyi and the Dog Aging Project Biobank at Cornell University in the US led by Dr. Marta Castelhano have been established to support the ongoing research at the beforementioned cohort studies. Previously, only a few instances of dog biobanking activities and related investigations had been reported in research papers, and consequently, the public awareness of the topic is very low. This may have an impact on the effectiveness of sample acquirement, similarly to what was shown for human biobanking [5]. Therefore, the first canine biobanks need to proactively make the concept more recognized - both among researchers and the general public.

The CBTB has already gained much from the expertise of the Senior Family Dog Project in managing owner contacts and distributing results through popular science forums. Dissemination is an important facet of companion dog research, which has to build its fundaments on the intent of dog owners to participate in projects. However, consenting to postmortem collection might be an entirely different issue than participating in behavioral experiments. Asking owners straightforwardly to make anticipatory considerations regarding the death of their beloved animal may not be the ideal way to increase participation. Therefore, communication strategies may require special approaches. It has been shown in human biobanking, that several factors may determine the motives of donation, and both education and public awareness seemed to play a pronounced role. Therefore, learning about how the donations would benefit others in the future can be an important motivator for donation. In addition, owners, who have already enrolled in follow-up studies, which test therapies and interventions [2,3] may also be more easily invited to consider a donation, as was shown in the case of specific study cohorts in human biobanking [5]. Both research groups maintaining a canine tissue bank have reached a great impact in popular science media and have already published results, which were based on samples taken from donated dogs [1,6].

Importantly, however, no specific methodological publications have been dedicated to this topic, in contrast to human biobanking, which has had its own literature developed. Setting the methodological standards and sharing experiences, therefore, should also be put in the focus during the advancement of the first canine biobanks [7]. Given that the Dog Aging Project Biobank has set its fundaments at Cornell University, which has already accumulated vast experience in the standardization of biobanking processes [8], it may take the lead in the development and validation of specific protocols for canine biobanking.

Altogether, the experiences gained, and results made within these institutes could lead the way of establishing a worldwide net of canine biobanks.
